# Preclinical evaluation of biomarkers associated with antitumor activity of MELK inhibitor

**DOI:** 10.18632/oncotarget.7685

**Published:** 2016-02-24

**Authors:** Suyoun Chung, Kyoko Kijima, Aiko Kudo, Yoshiko Fujisawa, Yosuke Harada, Akiko Taira, Naofumi Takamatsu, Takashi Miyamoto, Yo Matsuo, Yusuke Nakamura

**Affiliations:** ^1^ OncoTherapy Science, Inc., Kawasaki, Kanagawa, Japan; ^2^ Department of Medicine and Surgery, The University of Chicago, Chicago, IL, USA

**Keywords:** MELK, xenograft model, kinase inhibitor, molecular pharmacology, biomarker

## Abstract

MELK is upregulated in various types of human cancer and is known to be associated with cancer progression, maintenance of stemness, and poor prognosis. OTS167, a MELK kinase inhibitor, shows potent growth-suppressive effect on human tumors in a xenograft model, but the detailed mode of action has not been fully elucidated. In this study, we demonstrate the molecular mechanism of action of MELK inhibitor OTS167 in a preclinical model. OTS167-treated cells caused morphological transformation, induced the differentiation markers, and reduced stem-cell marker expression. Furthermore, we identified DEPDC1, known as an oncogene, as an additional downstream molecule of the MELK signaling pathway. MELK enhanced DEPDC1 phosphorylation and its stability. The expression of MELK and downstream molecules was decreased in OTS167-treated xenograft tumor tissues, which revealed central necrosis and significant growth suppression. Our data should further shed light on the mechanism of action how OTS167 suppresses tumor growth through the inhibition of the MELK signaling pathway and suggest the possibility of biomarkers for the assessment of clinical efficacy.

## INTRODUCTION

Cancer is the second most common cause of death in US in 2015 [1] and has been the leading cause of death in Japan since 1981[2]. Despite of the improvement in cancer treatment modalities, clinical outcome for some cancers such as triple-negative breast cancer (TNBC), pancreatic cancer and small cell lung cancer are still lagging behind others [3-5]. Although it causes severe adverse drug reactions, conventional chemotherapy is still the best therapeutic option and standard of care for these patients. Thus, the development of novel targeted therapeutics for these cancers are urgently needed.

We previously reported MELK (maternal embryonic leucine zipper kinase) as a potential and promising molecular target for development of novel cancer therapy [6]. Elevated MELK expression was reported in various types of human cancer [6-10] including hematological malignancies [11] with hardly detectable expression in normal organs except the testis. MELK expression was also associated with therapeutic resistance and poor prognosis [9, 12, 13]. To date, several proteins were identified as substrates or downstream molecules of the MELK pathway [6, 14, 15]. Through the analysis of MELK itself and these molecules, MELK was shown to be involved in many aspects of cancer traits, such as cell proliferation, anti-apoptosis and cell invasion. Furthermore, recent studies revealed upregulation of MELK in cancer stem cells and indicated MELK as a potential marker for cancer stem cells [15, 16]. Since targeting MELK alone or with other treatment modalities has a possibility to overcome therapeutic resistance by suppressing CSCs as well as cancer proliferation, many efforts have been paid to develop a MELK kinase inhibitor(s) [14, 17].

OTS167 was developed as a novel potent and selective MELK kinase inhibitor with strong antitumor activity in both solid and hematological cancers [11, 14], and is currently conducted a first-in-human clinical trial in solid tumor. Unlike the conventional chemotherapy, the use of maximum tolerated dose (MTD) and the measurement of bulk tumor volume are not always appropriate for the clinical evaluation of molecular targeted drugs [18]. To optimize and personalize the dosing for targeted agents, detailed molecular pathway and biomarkers that were affected by the drug should be elucidated.

In this study, we report the molecular mechanism of action of OTS167 in a cancer xenograft model. We demonstrate that OTS167-treated cancer cells or tumor tissues reduce MELK protein levels and a proliferation marker Ki67. Furthermore, we report DEPDC1 as a novel downstream molecule in the MELK-signaling pathway and that FOXM1, p21, Slug and Snail as well as DEPDC1 expression levels are affected by OTS167. These results support our therapeutic concept that OTS167 suppresses tumor growth through the inhibition of MELK pathway and imply that molecular changes in the MELK-signaling molecules might serve as biomarkers for treatment with a MELK inhibitor OTS167.

## RESULTS

### Suppression of MELK induces cell morphological change

**Figure 1:**
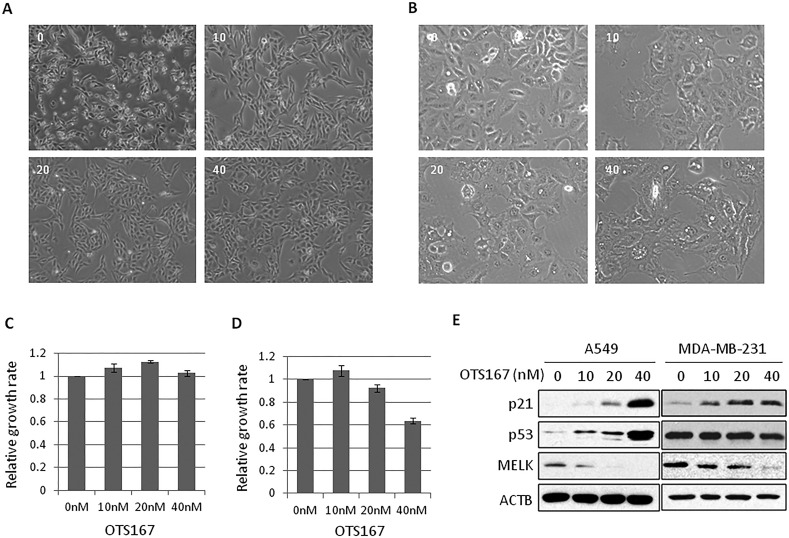
The morphological changes in OTS167-treated cancer cells **A.**, **B.** MDA-MB-231 (A) or A549 (B) cells were treated with OTS167 at a given concentration (nM). Cellular morphologies were observed at 24 hours after treatment. **C.**, **D.** The growth of OTS167-treated MDA-MB-231 (C) or A549 (D) cells. The proliferation assay were performed by an MTT assay to evaluate cell viability at 24 hours and graphed after standardization by control (0 nM) as 1.0. Error bars represent means ± SD of triplicates. **E.** The expression levels of p53 and p21 in OTS167-treated A549 (p53 wild-type) and MDA-MB-231 (p53 mutant) cells at 24 hours. ACTB was used as a protein-loading control.

To elucidate biological function of MELK, we treated cancer cells, MDA-MB-231 (breast cancer cell line) and A549 (lung cancer cell line), in which MELK was highly expressed, with a MELK inhibitor OTS167, and examined morphological changes of these cancer cells. As shown in Figure 1A and 1B, the morphology of MDA-MB-231 and A549 cells was drastically changed within 24 hours after the treatment with OTS167 even at 10-nM concentration. The cells appeared to be elongated in cytoplasmic projection. These morphological changes were also observed in other MELK-overexpressing cancer cells when they were treated with 20 nM of OTS167 for 24 hours (Supplementary Figure S1). Concordantly, the similar morphological changes were observed in MELK knocked-down cells (Supplementary Figure S2), supporting that these changes are likely to be caused through MELK suppression. Although no significant difference in the number of cells between the control and OTS167-treated groups was observed at this time point (Figure 1C and 1D), we found strong p53 and p21 induction in A549 cells with wild-type p53 (Figure 1E). Unexpectedly, although the level of p21 was higher in p53-wild-type A549 cells than in p53-mutated MDA-MB-231 cells, p21 protein was also increased in p53-mutant MDA-MB-231 cells, suggesting that induction of p21 seems to occur through the suppression of MELK activity regardless to the p53 status (Figure 1E). Interestingly, cancer cells with p53 wild-type showed relatively higher sensitivity to OTS167 than p53-mutant cells (Supplementary Figure S3, *p = 0.04*).

### MELK regulates DEPDC1 protein stability

**Figure 2:**
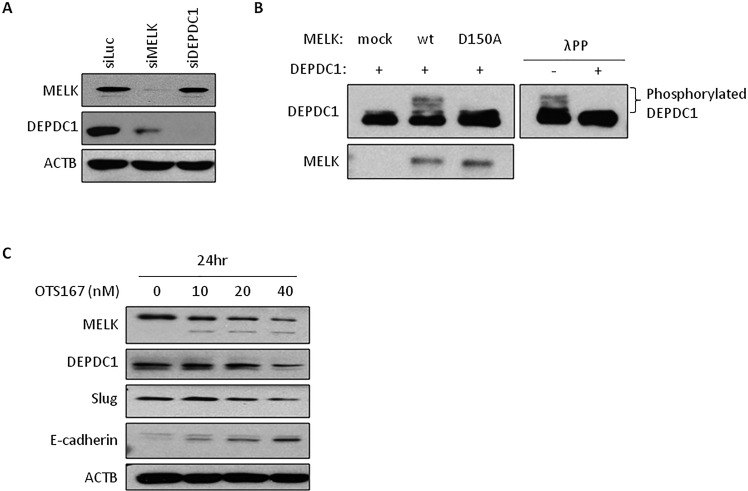
The expression of downstream molecules in OTS167-treated cells **A.** DEPDC1 expression in MELK knocked-down cells. Oligo siRNA for luciferase (control), MELK or DEPDC1 was transfected into MDA-MB-231 cells. After 24 hours of incubation, protein was detected by western blot analysis. siLuc; si-Luciferase. **B.** *In vivo* phosphorylation assay of DEPDC1. COS7 cells were co-transfected with DEPDC1 and either MELK (wt or D150A) or control mock vector. After 24 hours of incubation, cells were treated with okadaic acid for 3 hours. Proteins were separated by Phos-tag PAGE gel to detect phosphorylation of DEPDC1. For lambda phosphatase assay, proteins were incubated with lambda phosphatase before loading onto the gel. wt; wild-type MELK, D150A; kinase-dead mutant MELK, λPP; lambda phophatase. **C.** The expression of proteins in OTS167-treated cells. MDA-MB-231 cells were incubated with OTS167 for 24 hours at given concentration. MELK, DEPDC1, Slug, E-cadherin and ACTB were detected using specific antibodies. ACTB served as a protein-loading control.

We previously reported that MELK enhanced cell invasiveness through activation of DBNL [14], which is involved in the Rac/JNK signaling pathway [19]. Hence, we hypothesized that a part of MELK biological functions could be explained through its involvement in a small GTPase-signaling pathway. As DEPDC1 contains a Rho-GAP domain and is also upregulated in various types of human cancer, we investigated the relationship between MELK and DEPDC1, and found that the expression levels of MELK and DEPDC1 was strongly correlated in breast cancers as shown in Supplementary Figure S4A (Pearson's r = 0.6). We then examined DEPDC1 and MELK protein levels in cells treated with siRNA targeting MELK or DEPDC1. In MELK knocked-down cells, DEPDC1 protein level was significantly reduced without any change at the transcriptional level. On the other hand, no change was observed in MELK mRNA or protein level in the cells treated with siDEPDC1 (Figure 2A and Supplementary Figure S4B). We further performed an *in vivo* phosphorylation assay to examine phosphorylation status of DEPDC1. As shown in Figure 2B, DEPDC1 phosphorylation was enhanced in cells in which wild-type MELK was introduced, compared with cells transfected with control mock or kinase-dead MELK (D150A) vector. DEPDC1 phosphorylation was confirmed by disappearance of this band with phosphatase treatment (Figure 2B). These results have suggested that MELK is upstream of DEPDC1 and regulates DEPDC1 protein stability through its phosphorylation.

### MELK suppression induces loss of stem-like properties

MELK phosphorylates MELK itself and this autophosphorylation contributes to the MELK stability. When pharmacological inhibition of this autophosphorylation by OTS167 occurs, MELK protein is degraded rapidly (Figures 1E and 2C). Since MDA-MB-231 cells have an undifferentiated, cancer stem-like characteristics [20, 21], we examined the expression level of one of cancer stem cell markers, Slug (also known as Snail2), in OTS167-treated cells by western blot analysis, and found that Slug protein level was reduced with OTS167 treatment in a dose-dependent manner as similar to the MELK and DEPDC1 reduction (Figure 2C). Because Slug is also known to negatively regulate the E-cadherin expression, we examined E-cadherin protein level and confirmed its induction by the MELK inhibition in an OTS167 dose-dependent manner. These results indicated that MELK suppression reduced cancer stem cell population and might induce cell differentiation.

### OTS167 strongly induces antitumor activity in xenograft model

**Figure 3:**
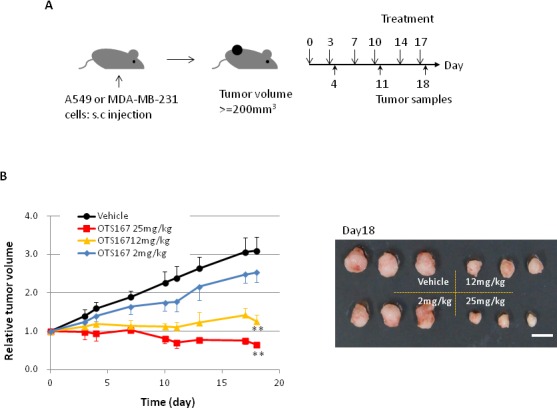

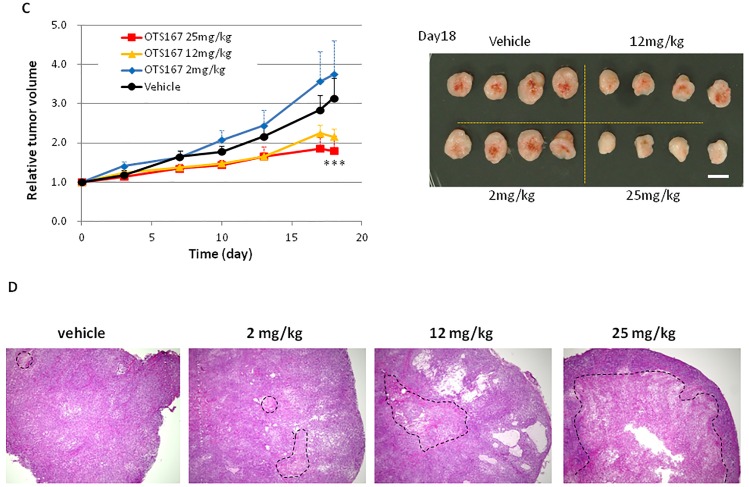
*In vivo* study of OTS167 **A.** Schematic presentation of the animal study. s.c injection; subcutaneous injection. Mice bearing A549 (B) or MDA-MB-231 (C) were treated with either vehicle or OTS167 at given concentration for 3 weeks. **B.** Relative tumor volume (left) and the representative images of tumors on day 18 (right) after treatment of A549 xenograft mice. Mean tumor volumes ± SD (*n* = 3 per each treatment group) are shown. **C.** Relative tumor volume (left) and the representative images of tumors on day 18 (right) after treatment of MDA-MB-231 xenograft mice. Mean tumor volumes ± SD (*n* = 4 per each treatment group) are shown. Scale bars in B and C equal 10mm. **p < 0.001, ***p = 0.02 by *t*-test. **D**. H&E staining of A549 tumor tissues at day 4 (original magnification: x 40). Necrotic regions are circumscribed by broken line.

We further performed animal xenograft experiments to examine the correlation between pharmacological effect and biomarker changes. A549 lung cancer cells or MDA-MB-231 breast cancer cells were inoculated into mice. After tumor sizes reached an average volume of 200 mm^3^, OTS167 or vehicle was administered intravenously twice a week for 3 weeks (Figure 3A). Tumor growth was significantly suppressed in the OTS167-treatment group of the A549 model in a dose-dependent manner. Tumor growth inhibition (TGI) in the group treated with 2, 12, or 25 mg/kg of OTS167 in A549 xenograft mice was 27, 88, and 117%, respectively (Figure 3B). In MDA-MB-231 xenograft mice, tumor suppressive effect of OTS167 was not as strong as that against A549 cells, but modest levels of growth suppressive effect was observed at the doses of 12 and 25 mg/kg with TGI of 51 and 66 %, respectively (Figure 3C). To further elucidate the cellular and molecular changes in OTS167-treated tumor tissues, we collected xenograft tissues on day 4, 11, and 18, and performed western blot analysis and immunohistochemical analysis. H&E staining of tumor tissues clearly revealed massive central necrosis even in an early time-point (day 4) after the treatment as shown in Figure 3D and Supplementary Figure S5. Necrotic areas became larger in an OTS167 dose-dependent manner, suggesting OTS167 induces early intratumoral changes without decrease in tumor volume. The administration of OTS167 was well tolerated in xenograft model without any significant toxicity and body weight loss (Supplementary Figure S7).

### Alterations of MELK pathway is correlated with OTS167 antitumor effects

**Figure 4:**
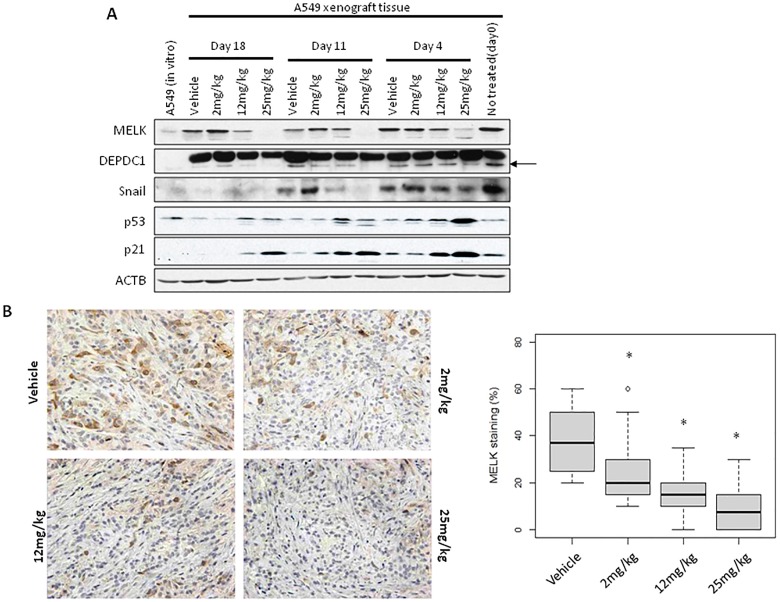

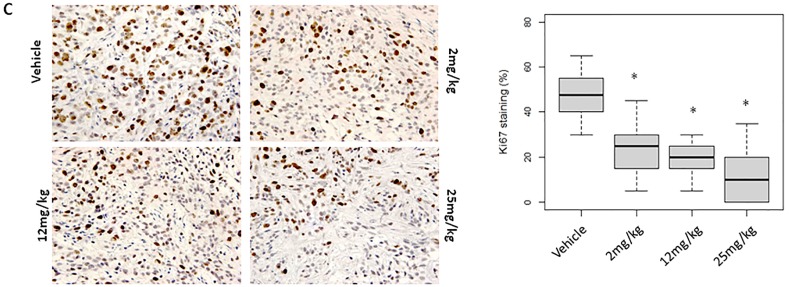
MELK and Ki67 expression in OTS167-treated tumor tissue **A.** Western blot analysis of MELK, DEPDC1, Snail, p21 and p53 in A549 xenograft tumor tissues. ACTB served as a protein-loading control. Arrow indicates DEPDC1. **B.**, **C.** Immunohistochemistry using tumor tissues collected on day 4 after OTS167 treatment (original magnification: x 400). Tissues were stained using anti-MELK antibody (B) or anti-Ki67 antibody (C). Box plots represent the percentage of positive cells stained with each antibody. Horizontal lines represent mean and error bars indicating the interquartile ranges of 30 ROIs per group.*p < 0.0001 by ANOVA and *t*-test.

**Figure 5:**
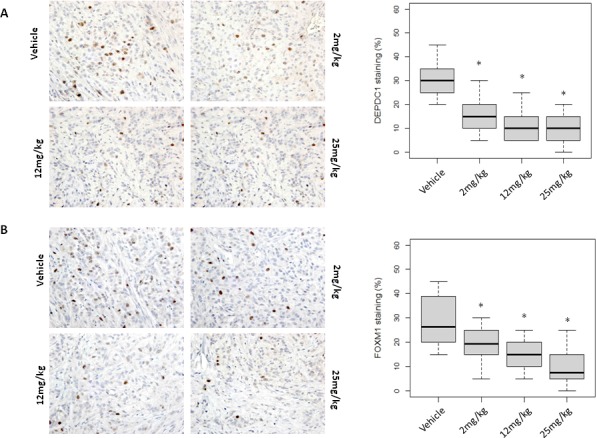

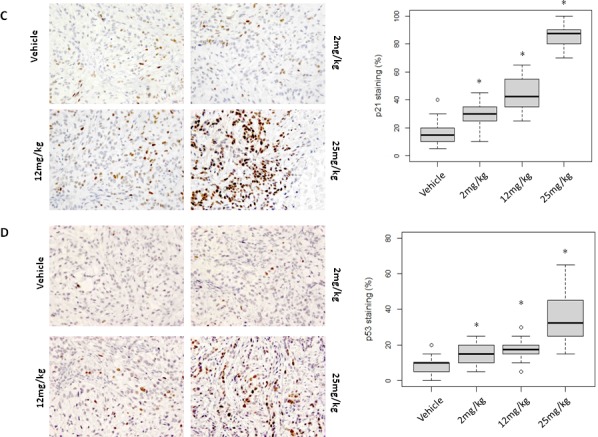
Molecular changes in OTS167-treated tumor tissue Immunohistochemical analysis using xenograft tissue collected on day 4. **A.** DEPDC1, **B.** FOXM1, **C.** p21, and **D.** p53 were examined (original magnification: x 400). Box plots represent the percentage of positive cells stained with each antibody. Horizontal lines represent mean and error bars indicating the interquartile ranges of 30 ROIs per group. *p < 0.0001 by ANOVA and t *t*-test.

To elucidate applicability of MELK protein levels as a pharmacodynamic biomarker, we firstly performed western blot analysis using A549 and MDA-MB-231 xenogarft tissues. Xenograft tissues from mice administered 12mg/kg or 25mg/kg on day 11 and 18 showed significant decrease of MELK protein (Figure 4A and Supplementary Figure S6A). Concordantly, immunohistochemical analysis showed decrease of MELK protein; the proportion of an area with MELK-positive cells was significantly reduced in a dose-dependent manner (*p < 0.0001*, Figure 4B and Supplementary Figure S6B, C). In addition, we examined a proliferation marker Ki67 by immunohistochemistry and found that its positivity was significantly decreased in OTS167-treated tumors (*p < 0.0001*, Figure 4C), compared with the control group. These data provided the evidence supporting that impaired tumor growth was caused by the suppression of MELK activity. Furthermore, we performed western blot and immunohistochemical analyses of MELK-downstream molecules. The expression levels of DEPDC1 and FOXM1 were significantly decreased in MELK-inhibitor treated tissues (Figures 4A and 5A, 5B, *p < 0.0001*). Interestingly, p21 and p53 expression levels were also significantly increased in OTS167-treated tissues (Figures 4A and 5C, 5D, *p < 0.0001*). However, interestingly, the expression of p21 protein was slightly induced at day 4 in MDA-MB-231 xenograft tissues in which p53 was mutated, but reduced at later days (Supplementary Figure S6A). Moreover, expression levels of Snail (A549, Figure 4A) and Slug (MDA-MB-231, Supplementary Figure S6A) were also drastically decreased in tumor tissues treated with the MELK inhibitor, indicating that targeting MELK could reduce cancer stem cell population in tumor tissues.

## DISCUSSION

The remarkable technical advances in molecular biology as well as genetics/genomics have allowed us to effectively identify disease-specific and/or disease-causing molecular alterations. Accordingly, there is a striking paradigm shift in cancer treatment from ‘one-size-fits-all’ to the selection of therapy based upon patient's molecular abnormalities, so called ‘personalized or precision cancer medicine’ [22]. To achieve the goal of ‘cancer precision medicine’, co-development of molecular-targeted drugs together with molecular diagnostics is critically important. Not only the selection of appropriate targets, but also the elucidation of molecular mechanisms of targets is essential to deliver drugs effectively and safely to patients [23].

OTS167 was developed as a MELK kinase inhibitor with potent and selective antitumor activity without any obvious toxicity at the effective dose in our preclinical study. *MELK* is specifically and frequently upregulated in various types of human cancer including breast cancer, brain tumors, prostate cancer, and acute myeloid leukemia [6-9, 11]. The mechanism of *MELK* transactivation is not yet elucidated though several research groups and the public database such as COSMIC and TCGA indicated that there was no genetic alteration such as gene amplification or epigenetic dysregulation [24-26]. We assume that since MELK is highly expressed in stem cells and cancer cells are considered to originate from stem cells, cancer tissues including a relatively high proportion of cancer stem cells reveal higher MELK expression. In addition to the overexpression, this kinase is shown to have important biological roles in cancer cells such as proliferation, survival, metastasis and maintenance of stemness. Several molecules were identified as substrates of MELK or downstream proteins in the MELK pathway [6, 14, 15, 27]. However, the biological function of MELK in cancer cells is still far from full understanding.

In the present study, we have performed further biological analysis of MELK protein as well as pharmacologic and pharmacodynamic evaluation of OTS167. We found that suppression of MELK induced enlargement of cytoplasm and induction of cytoplasmic projection like a neuronal-cell phenotype. These phenotypes were not observed in cells treated with cytotoxic agents like adryamycin or paclitaxel. Because MELK promoted cell invasiveness [10, 14], it was almost certain that MELK is involved in regulation of cytoskeleton. DEPDC1 is also upregulated in several types of human cancer including breast cancer according to the public database [28], and a recent study indicated that DEPDC1 is related to cytoskeletal regulation [29]. Hence, we examined the relationship between MELK and DEPDC1, and found that MELK is likely to regulate phosphorylation and stability of DEPDC1 protein. Intriguingly, DEPDC1 was reported as a gene that is associated with breast cancer brain metastasis [30]. Though further investigation is needed, our results suggest that MELK might be involved in brain metastasis of breast cancer through DEPDC1 regulation.

In addition, MELK expression was also elevated in cancer stem cells and overexpression of MELK was shown to enhance spheroid formation of cancer cells [15, 16, 31]. We previously investigated the suppression of breast cancer spheroid formation by pharmacological inhibition of MELK [14]. OTS167 was more potently inhibited the mammosphere formation rather than proliferation of attached cancer cells. In this study, we analyzed the expression of stem-cell marker using OTS167-treated cell lines or xenograft tissues. Slug and Snail, well known markers for stem cells, were indicated to induce EMT (epithelial-mesenchymal transition) [32, 33]. We found that expression of MELK and stem cell marker proteins was higher in *in vivo* A549 xenograft tissues than *in vitro* cultured cells (Figure 4A), further suggesting previous results that cancer stem cells expressing MELK at high level have the growth advantage *in vivo*. We clearly demonstrated that the expression of Slug and Snail proteins was decreased in tumors which were treated with OTS167, indicating that OTS167 might lead to loss of cancer stem-like characteristics through inhibition of MELK function.

Not only cancer stem cell markers, but other MELK-related proteins such as DEPDC1, and FOXM1 as well as major tumor-suppressive proteins, p21 and p53, also showed drastic alterations in their protein levels with treatment of OTS167 both in cell lines and in xenograft tumor tissues. It is reported that suppression of MELK by siRNA activated the p53 pathway and induced cell cycle arrest [34], and our results also indicated the strong activation of p53 and p21 in p53 wild-type cancer cells with treatment of MELK inhibitor. However, the activation of p21 was also observed in p53-mutant cells although it was relatively weak in p53-mutated cancer cells. These data indicated that p21 might be activated by MELK suppression regardless to the p53 status. As described above, cancer cells with p53 wild-type showed relatively higher sensitivity to OTS167 than p53-mutant cells. Although further validation is required, MELK inhibition may be enhanced in the presence of wild-type p53 since p53 can activate multiple downstream genes involved in growth arrest or apoptosis [35]. Moreover, changes in the levels of proteins examined here were observed even at an early time-point of the treatment and could be used for monitoring the antitumor effect. Thus, the clinical efficacy in the patients who will be treated with OTS167 might be predictable by the analysis of these markers in biopsy samples at the relatively early stage of treatment.

In conclusion, our findings provide further evidence of mode of mechanism that OTS167 suppresses tumor growth by impairment of the MELK signaling pathway. Although further evaluation in human cases is required, the baseline changes of protein expression can be applicable to assess the clinical response of OTS167 as pharmacodynamic biomarker. Moreover, it would be possible to determine optimal biological dosage and the best treatment strategy of OTS167 in patients using these biomarkers.

## MATERIALS AND METHODS

### Cell lines

MDA-MB-231 and A549 cells were purchased from ATCC (USA). COS7 cell was purchased from RIKEN BRC Cell Bank (Japan). Cells were authenticated by microscopic morphology check prior to perform each experiment and screened for mycoplasma contamination by PCR-based detection kit (Takara). Cells were cultured under appropriate media recommended by suppliers with 10% FBS and 1% antibiotic-antimycotic solution (Wako). All cells except MDA-MB-231 were maintained at 37°C in humidified air with 5% CO_2_. MDA-MB-231 was maintained at 37°C in humidified air without CO_2_.

### Cell morphology assessment and MTT assay

Cells were plated onto culture dish and treated with DMSO or OTS167. Cell morphology was examined at 24 or 48 hours after OTS167 treatment using digital camera connected with Olympus phase-contrast microscope. For MTT assay, cell viability was measured by using Cell-Counting Kit-8 (Dojindo) according to manufacturer's instructions. Absorbance at 450 nm (630 nm as reference) was measured with the iMark microplate reader (BioRad).

### Antibodies and reagents

Following antibodies were used in this study: Anti-human MELK antibody (in-house, previously described [14]), anti-human DEPDC1 (in-house, previously described [36]), anti-human FOXM1, anti-human p53 (Santa Cruz Biotechnology), anti-human Ki67 (Millipore), anti-human p21, anti-human Slug, anti-human Snail (Cell Signaling Technology), anti-human E-cadherin (BD biosciences), anti-HA (Roche), anti-FLAG M2, and anti-beta-actin antibodies (Sigma). OTS167 was dissolved in DMSO (Sigma) for cell culture or in 5% glucose solution for animal study. The target sequences of oligo siRNAs were 5′-CUUACGCUGAGUACUUCGAUU-3′ for MELK and 5′-AGUUCAUUGGAACUACCAAUU-3′ for Luciferase (control). DEPDC1 oligo siRNA was purchased from Santa Cruz Biotechnology (sc-78918).

### Immunoblotting

Cells were lysed with RIPA buffer (25mM Tris pH 7.6 with 150mM NaCl, 1% NP-40, 1% sodium deoxycholate, and 0.1% SDS) containing protease inhibitor cocktail (Calbiochem), Na_3_VO_4_ and NaF (Nacalai Tesque). Xenograft tissues were frozen in liquid nitrogen and ground to a fine powder, and then the powder was dissolved in RIPA buffer to extract protein. Cell or tissue lysates were collected after centrifugation and analyzed protein concentration using BCA Protein Assay kit (Thermo Scientific) according to manufacturer's protocol. Then proteins were separated by electrophoresis using 8% or 15% SDS-PAGE gel and subsequently transferred onto nitrocellulose or PVDF membrane. Membranes were incubated with the first antibody as described above. After washing, membranes were incubated with horseradish peroxidase (HRP)-conjugated secondary antibody for 1 hour at room temperature and then developed using ECL Western Blotting Detection Reagents (GE Healthcare).

### *In vivo* phosphorylation assay and Lambda protein phosphatase assay

Detailed information of plasmids expressing FLAG-tagged DEPDC1 or HA-tagged MELK (wild-type (wt) or kinase-dead mutant (D150A)) is described in previous reports [6, 36]. DEPDC1 was co-transfected with mock, MELK wild-type or MELK D150A into COS7 cells using X-tream Gene HP (Roche). After 24-hour incubation, cells were treated with 0.2uM Okadaic acid (Sigma) for 3 hours and collected with RIPA buffer. The samples were then separated by 12.5% SuperSep Phos-tag gel (Wako) electrophoresis. For lambda phosphatase assay, lambda phosphatase (NEB) was added into cell lysate and incubated for 1 hour at 30°C. Proteins were separated by SuperSep Phos-tag gel and transferred onto nitrocellulose membrane. Membrane was incubated with anti-Flag antibody as described above. After washing, membranes were incubated with horseradish peroxidase (HRP)-conjugated secondary antibody for 1hour at room temperature, developed using ECL Western Blotting Detection Reagents (GE Healthcare).

### Immunohistochemistry

Each excised xenograft tumor was fixed by formaldehyde, embedded in paraffin, and then sliced on glass slides. For immunohistochemistry, paraffin-embedded tumor section was deparaffinized, rehydrated, stained with hematoxylin (Dako) and eosin (Sakura Finetek), and analyzed by IHC using antibodies as described above. Briefly, the tumor sections were treated with xylene and ethanol, and incubated with Antigen Retrieval Solution pH9 (Nichirei) to retrieve antigens. Then, tumor sections were incubated with 3% H_2_O_2_ solution (Wako) for peroxidase blocking and Serum-free Protein Blocking Reagent (Dako), according to manufacturer's instructions. These sections were incubated with primary antibodies diluted in the Antibody Diluent Solution (Dako), followed by incubation with Histofine Simple Stain MAX PO (Nichirei), and then stained with substrate-chromogen (Histofine DAB kit, Nichirei). Finally, tumor sections were counterstained with hematoxylin (Dako) and observed in a bright-field microscope (Leica).

### Image analysis of immunohistochemistry

All images were taken with Leica DM2000 microscope and IM50 software (Leica). Thirty regions of interest (ROIs) per group were randomly selected from each of stained sections and the ratio of stained cells (DAB-positive)/hematoxylin-positive nuclei in ROI was quantified. The mean positive rate was graphed by R statistical environment version 3.2.0 [37].

### Animal study

The animal experiments were conducted in accordance with Institutional Guidelines for the Care and Use of Laboratory Animals. A549 or MDA-MB-231 cells were injected into NOD-scid mice (Charles River Laboratory). When xenografts had reached an average volume of 200 mm^3^, animals were randomly sorted into groups (9 mice each/group for an A549 xenograft model and 12 mice each/group for a MDA-MB-231 model). For intravenous administration, OTS167 was formulated in 5% glucose and injected into the tail vein twice weekly for 3 weeks. Each xenograft mice was treated with OTS167 or vehicle of given concentration at days 0, 3, 7, 11, 13 and 17. Tumor volumes were determined twice per week using a caliper. The results were converted to tumor volume (mm^3^) by the formula length × width^2^ × 1/2. Tumor growth inhibition (TGI) was calculated according to the formula {1-(T-T_0_)/(C-C_0_)} × 100, where T and T_0_ are the mean tumor volumes at days 18 and 0, respectively, for the OTS167-treated group, and C and C_0_ are those for the vehicle control group. Three or four mice of each group were sacrificed at days 4, 11 and 18 to collect tumors.

### Statistical analysis

Statistical analyses and plots were carried out using R statistical environment version 3.2.0 [37]. All values were presented as means ± SD. Student's *t*-tests were used for two-group comparison. For multiple-group comparison, one-way analysis of variance (ANOVA) method with Tukey's *post hoc* corrections was used. The level of significance was set at *p < 0.05*.

## SUPPLEMENTARY MATERIAL FIGURES


