# Correction: Preclinical evaluation of biomarkers associated with antitumor activity of MELK inhibitor

**DOI:** 10.18632/oncotarget.27755

**Published:** 2020-10-13

**Authors:** Suyoun Chung, Kyoko Kijima, Aiko Kudo, Yoshiko Fujisawa, Yosuke Harada, Akiko Taira, Naofumi Takamatsu, Takashi Miyamoto, Yo Matsuo, Yusuke Nakamura

**Affiliations:** ^1^ OncoTherapy Science, Inc., Kawasaki, Kanagawa, Japan; ^2^ Department of Medicine and Surgery, The University of Chicago, Chicago, IL, USA

**This article has been corrected:** During image assembly, incorrect data was mistakenly used in Figure 5A, resulting in a duplicate image of the bottom two panels. The corrected Figure 5A is shown below. The authors declare that these corrections do not change the results or conclusions of this paper.

Original article: Oncotarget. 2016; 7:18171–18182. 18171-18182. https://doi.org/10.18632/oncotarget.7685

**Figure 5:**
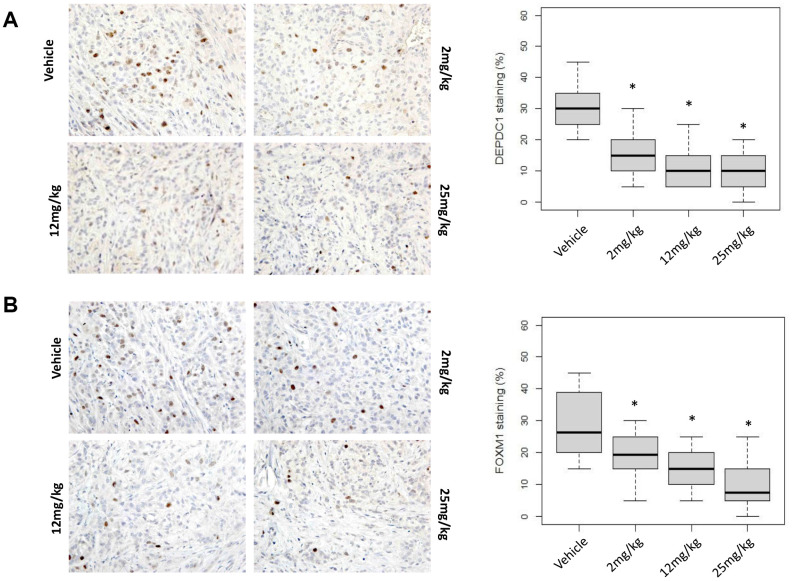
Molecular changes in OTS167-treated tumor tissue. Immunohistochemical analysis using xenograft tissue collected on day 4. (**A**) DEPDC1, (**B**) FOXM1, (**C**) p21, and (**D**) p53 were examined (original magnification: x 400). Box plots represent the percentage of positive cells stained with each antibody. Horizontal lines represent mean and error bars indicating the interquartile ranges of 30 ROIs per group. ^*^ *p* < 0.0001 by ANOVA and t *t*-test.

