# Supplementary material for: Preclinical evaluation of biomarkers associated with antitumor activity of MELK inhibitor
Source: Oncotarget. 2016 Feb 24;7(14):18171–82. doi: 10.18632/oncotarget.7685 (PMC4951280; doi:10.18632/oncotarget.7685)
Supplement: Supplementary file 1 [file oncotarget-07-18171-s001.pdf]

Preclinical evaluation of biomarkers associated with antitumor activity of MELK inhibitor

Supplementary Material

Materials and Methods

Cell lines. Nineteen human breast cancer cell lines and 32 human lung cancer cells used for anti-proliferative assay with OTS167 were as follows; HCC1500, DU4475, T47D, MDA-MB-453, SK-BR-3, ZR-75-1, BT-474, BT-549, MDA-MB-468, BT-20, MDA-MB-231, MDA-MB-157, HCC70, MCF7, HCC1937, HCC1143, HCC1395, BT-483, A427, A549, DMS114, NCI-H460, NCI-H522, NCI-H1373, NCI-H23, SK-LU-1, NCI-H520, NCI-H358, NCI-H1435, SK-MES-1, NCI-H226, SW1573, H69AR, NCI-H1650, NCI-H1666, NCI-H1781, NCI-H2170, SW900, NCI-H596, NCI-H1793 (purchased from ATCC), YMB-1, EBC-1, LU99, PC-3, SBC3, SBC5, VMRC-LCD (purchased from JCRB), PC-14 (purchased from RIKEN), LC176, DMS273, and LC174 (kindly provided by Dr. Y. Nakamura, the University of Chicago). Cells were authenticated by microscopic morphology check prior to perform each experiment and screened for mycoplasma contamination by PCR-based detection kit (Takara). Cells were cultured under appropriate media recommended by suppliers with 10% FBS and 1% antibiotic-antimycotic solution (Wako). All cells except MDA-MB-231, SW900 and SW1573 were maintained at 37°C in humidified air with 5% CO_2. MDA-MB-231, SW900 and SW1573 cells were maintained at 37°C in humidified air without 5% CO_2.

Anti-proliferative assay. Cells were seeded into 96-well culture plates and treated with OTS167 for 72 hours at 37°C. Cell viability was measured by the colorimetric assay using Cell Counting Kit-8 (Dojindo) and plates were read using a spectrophotometer at a wavelength of 450 nm. IC50 values were calculated by nonlinear four parameter fit using SigmaPlot, version 10.0 (Systat Software, Inc.).

Semi-quantitative RT-PCR. Total RNAs were purified using RNeasy Mini kit (QIAGEN) according to the manufacturer's protocol. Subsequently, reverse transcription was carried out using SuperScript II transcriptase (Thermo Fisher) with oligo-dT primer (Roche). PCR was performed with primers as follows; 5'-GTGGCTTTGCAAAGGTCAAA-3' and 5'-AGCTGATTGACTTTGGTCTCT-3' for *MELK,* 5'-GATCTCCCTGAACCTCTACTTAC-3' and 5'-ATCGATGGCAACCCTCTCTAAATG-3' for *DEPDC1*, 5'-AGGATGCAGAAGGAGATCAC-3' and 5'-AGTTGCGTTACACCCTTTCT-3' for *β-actin* (*ACTB)*. *ACTB* gene was used as an internal control.

Statistical analysis. Statistical analyses and plots were carried out using R statistical environment version 3.2.0. All values were presented as means ± SD. Student's t-tests were used for two-group comparison. For multiple-group comparison, one-way analysis of variance (ANOVA) method with Tukey's *post hoc* corrections was used. The level of significance was set at $p<0.05$.

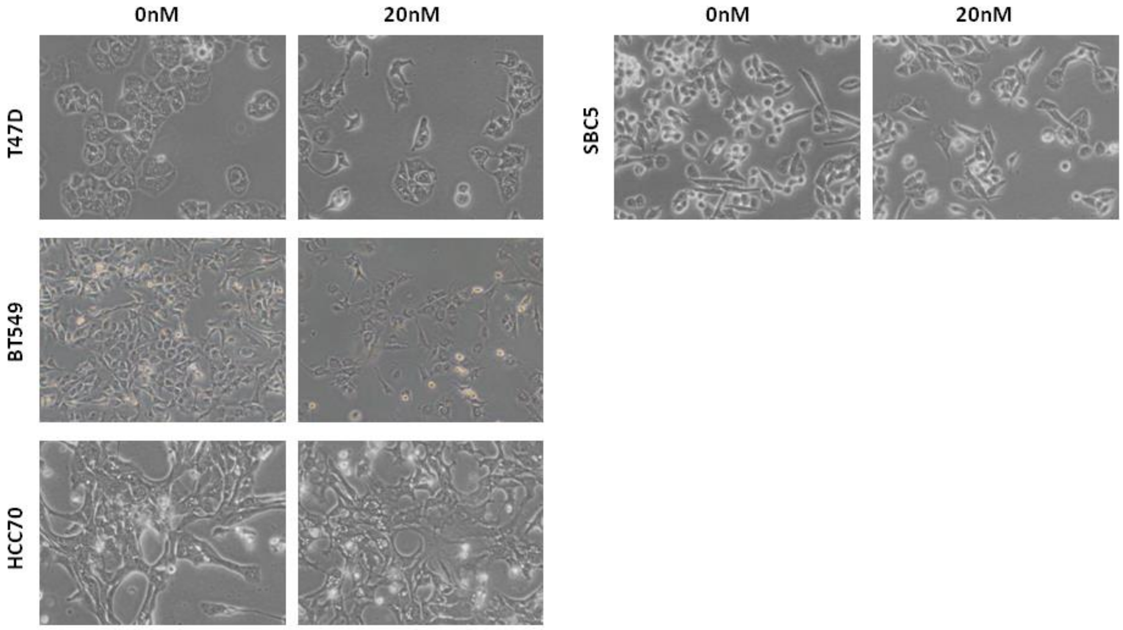

Supplementary Figure 1. Morphological changes of cancer cells after treatment with OTS167.

Breast (T47D, BT549, HCC70) or lung (SBC5) cancer cells were treated with DMSO (0nM) or OTS167 (20nM) for 24 hours. After treatment of OTS167, cells showed cytoplasmic projection and enlarged cytoplasm.

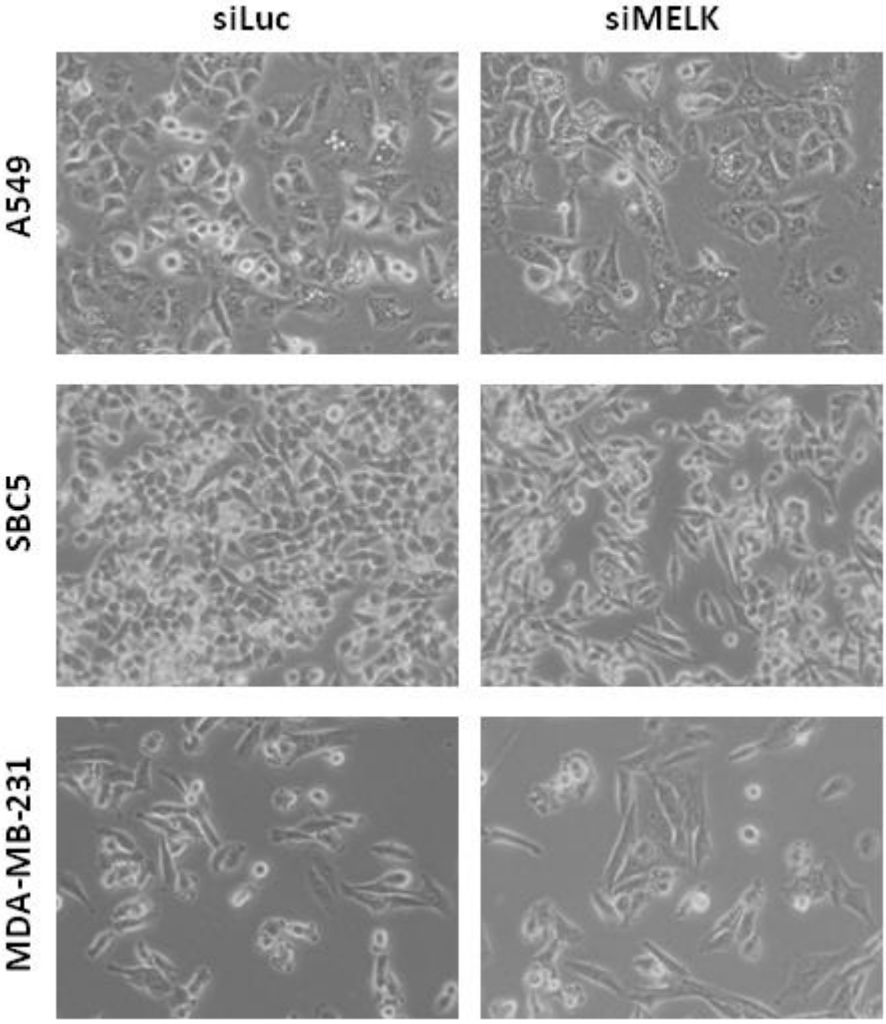

Supplementary Figure 2. Morphological changes in MELK knocked-down cells.
A549, SBC5 or MDA-MB-231 cells were transfected with oligo siLuc or siMELK. After 48 hours, cell morphology was observed by light microscopy. siLuc; si-Luciferase.

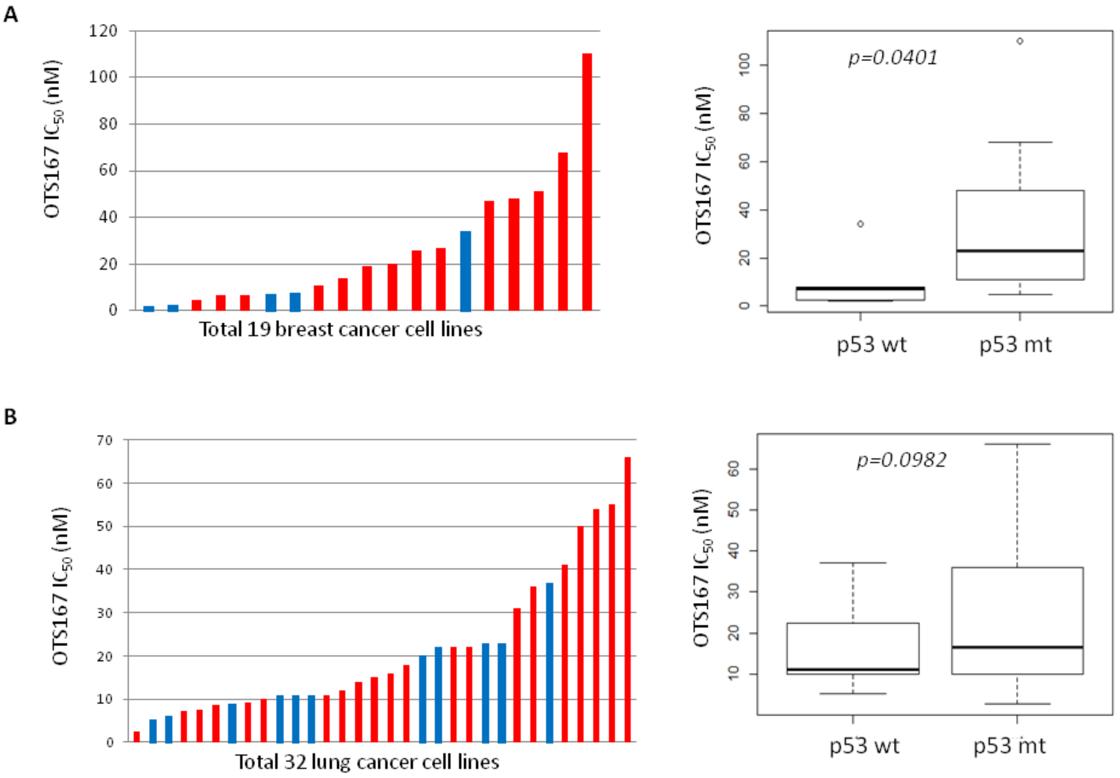

Supplementary Figure 3. Correlation between OTS167 sensitivity and p53 status in cancer cells.

(A, B) The relationship between OTS167 IC_{50} value (nM) and p53 status in breast cancer cell lines (A) and lung cancer cell lines (B). Blue bars indicate p53 wild-type cancer cell lines and red bars indicate p53-mutant cancer cell lines (right figures). Cells were grouped according to p53 status (wild-type; wt or mutant; mt). Box plots represent the IC_{50} value (nM) of cells and horizontal lines represent the mean. Error bars indicating the inter-quartile ranges of each group.

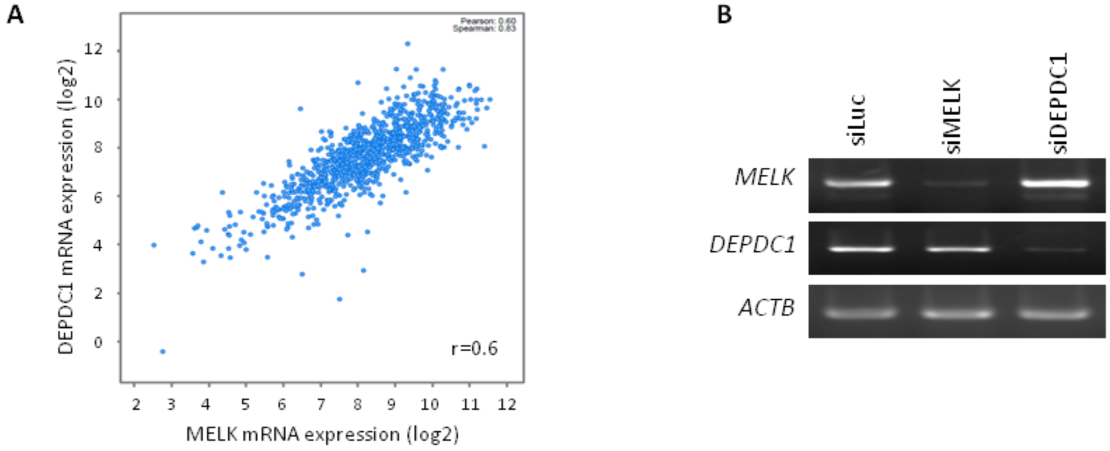

Supplementary Figure 4. Transcriptional expression of MELK and DEPDC1.

(A) Correlation of MELK and DEPDC1 expression in breast cancer. TCGA data of total 974 breast cancer samples was analyzed using the cBioPortal website (http://www.cbioportal.org/public-portal/). Pearson's r=0.6. (B) Oligo siRNA for luciferase, MELK, or DEPDC1 was transfected into MDA-MB-231 cells and incubated for 24 hours. After reverse transcription, PCR was performed to examine the transcriptional expression of MELK or DEPDC1.

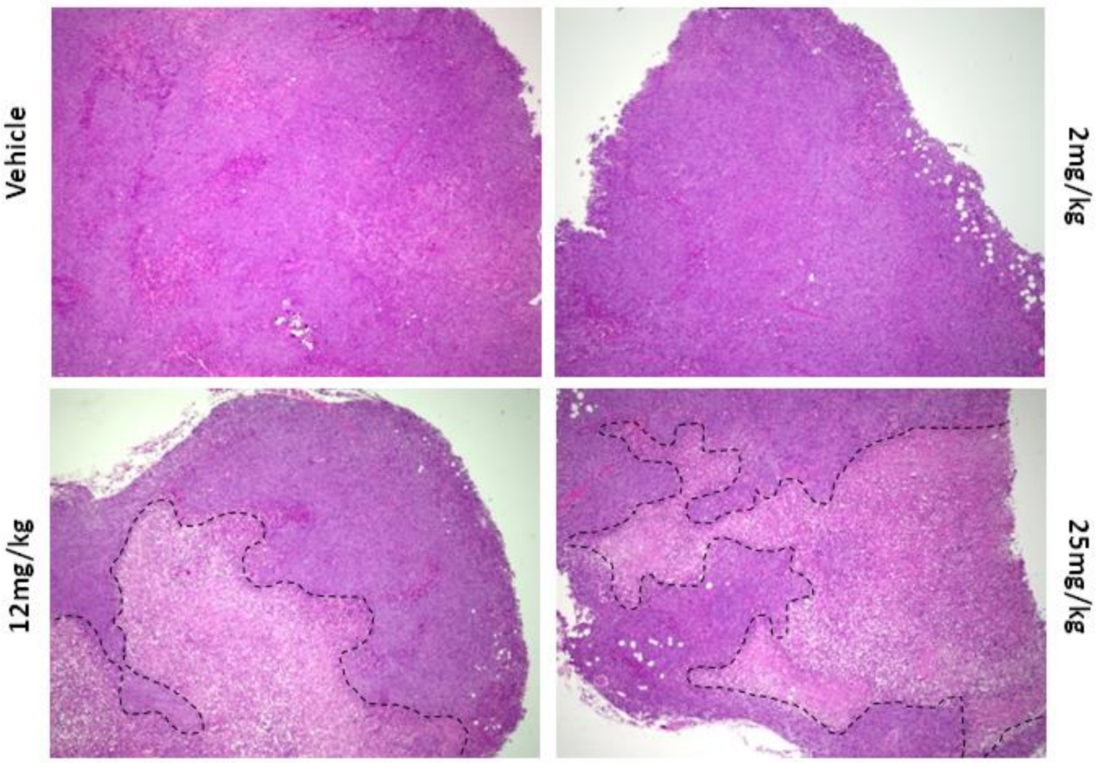

Supplementary Figure 5. H&E staining of MDA-MB-231 xenograft tissues.

H&E staining of MDA-MB-231 tumor tissues at day 4 (original magnification: x 40). Necrotic regions are circumscribed by broken line.

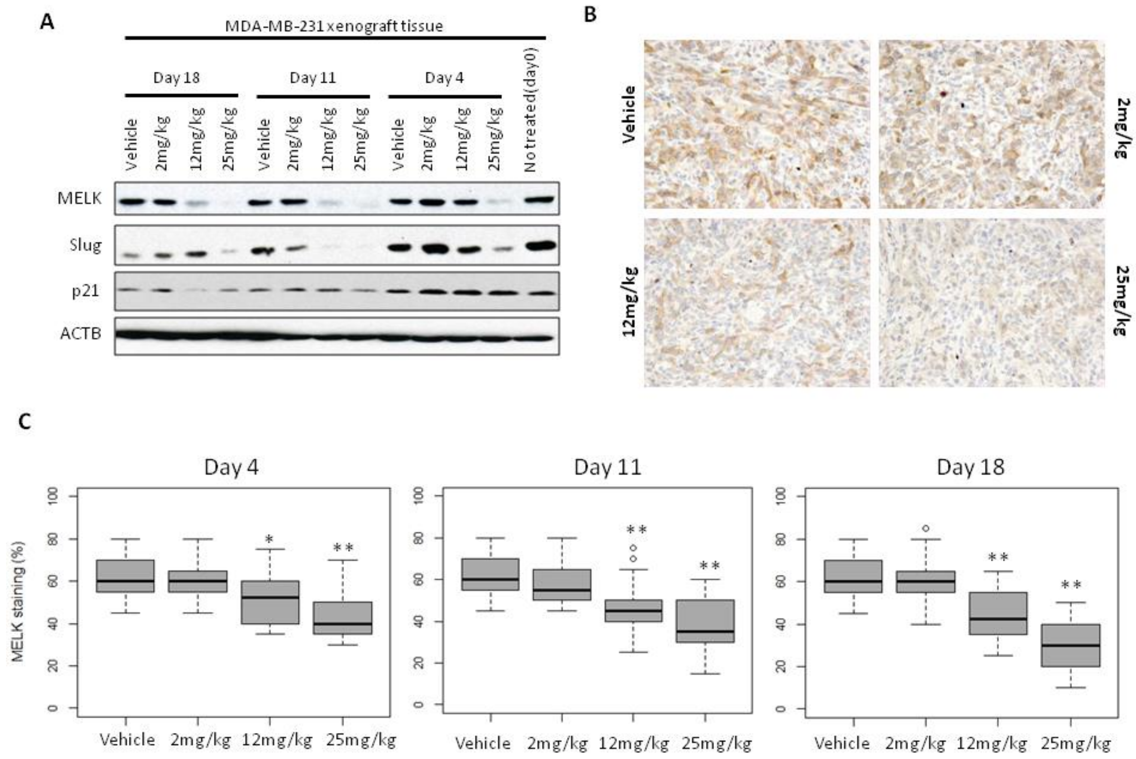

Supplementary Figure 6. Molecular changes in OTS167-treated MDA-MB-231 xenograft tissues.

(A) Western blot analysis for MELK, Slug and p21 proteins using MDA-MB-231 xenograft tissues. ACTB served as protein-loading control. The expression of MELK and Slug expression was significantly decreased in xenograft tissues from mice treated with 12mg/kg or 25mg/kg. (B) Immunohistochemical analysis of MELK using xenograft tissue collected on day 4 (original magnification: x 400). (C) Box plots represent the percentage of MELK positive cells on days 4, 11, and 18. Horizontal lines represent mean and error bars indicating the interquartile ranges of 30 ROIs per group. *$p=0.01$, **$p<0.001$ by ANOVA and t-test. Proportion of MELK-positive cells in 12mg/kg or 25mg/kg of OTS167-treated tissues was decreased in dose-dependent and time-dependent manners.

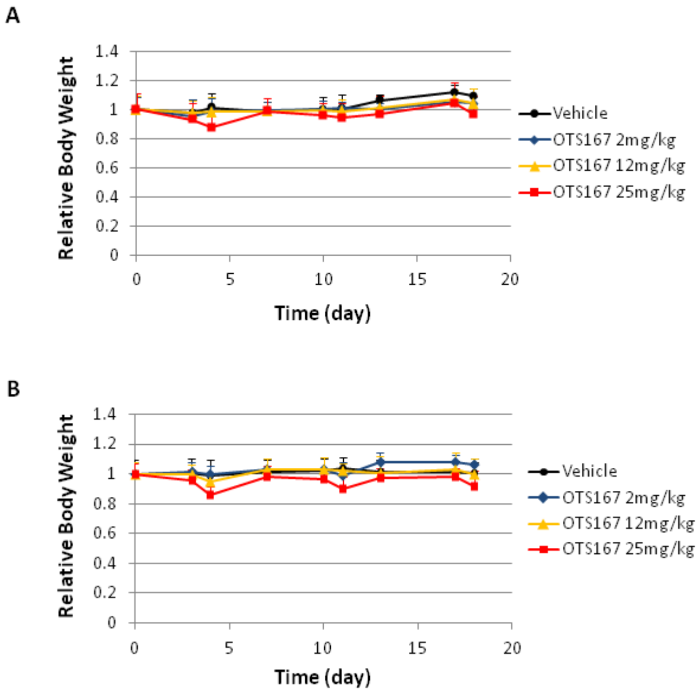

Supplementary Figure 7. Effect of OTS167 on body weights of xenograft mice.
Relative mean body weights ± SD in comparison with the mean body weight just before the administration (Day 0) in A549 (A, n=3 per each treatment group) or MDA-MB-231 (B, n=4 per each treatment group) xenograft model.
